# Superimposed Skilled Performance in a Virtual Mirror Improves Motor Performance and Cognitive Representation of a Full Body Motor Action

**DOI:** 10.3389/frobt.2019.00043

**Published:** 2019-06-21

**Authors:** Felix Hülsmann, Cornelia Frank, Irene Senna, Marc O. Ernst, Thomas Schack, Mario Botsch

**Affiliations:** ^1^Computer Graphics and Geometry Processing, Bielefeld University, Bielefeld, Germany; ^2^Social Cognitive Systems, Bielefeld University, Bielefeld, Germany; ^3^Neurocognition and Action, Bielefeld University, Bielefeld, Germany; ^4^Applied Cognitive Psychology, Ulm University, Ulm, Germany

**Keywords:** action observation, imitation learning, motor cognition, motor training, virtual reality, visual feedback, augmented feedback

## Abstract

Feedback is essential for skill acquisition as it helps identifying and correcting performance errors. Nowadays, Virtual Reality can be used as a tool to guide motor learning, and to provide innovative types of augmented feedback that exceed real world opportunities. Concurrent feedback has shown to be especially beneficial for novices. Moreover, watching skilled performances helps novices to acquire a motor skill, and this effect depends on the perspective taken by the observer. To date, however, the impact of watching one's own performance together with full body superimposition of a skilled performance, either from the front or from the side, remains to be explored. Here we used an immersive, state-of-the-art, low-latency cave automatic virtual environment (CAVE), and we asked novices to perform squat movements in front of a virtual mirror. Participants were assigned to one of three concurrent visual feedback groups: participants either watched their own avatar performing full body movements or were presented with the movement of a skilled individual superimposed on their own performance during movement execution, either from a frontal or from a side view. Motor performance and cognitive representation were measured in order to track changes in movement quality as well as motor memory across time. Consistent with our hypotheses, results showed an advantage of the groups that observed their own avatar performing the squat together with the superimposed skilled performance for some of the investigated parameters, depending on perspective. Specifically, for the deepest point of the squat, participants watching the squat from the front adapted their height, while those watching from the side adapted their backward movement. In a control experiment, we ruled out the possibility that the observed improvements were due to the mere fact of performing the squat movements—irrespective of the type of visual feedback. The present findings indicate that it can be beneficial for novices to watch themselves together with a skilled performance during execution, and that improvement depends on the perspective chosen.

## Introduction

Feedback is essential for skill acquisition as it delivers performance-related information and can help to identify potential errors and to make corrections needed for performance improvement (Magill, 2001; Magill and Anderson, 2012). While task-intrinsic feedback relates to information available as a result of task execution, augmented feedback is used to convey any kind of extra information in addition to task-intrinsic feedback. In sport settings, for instance, when it comes to learning a new motor skill that requires the execution of complex full body movements, looking at a mirror offers visual feedback or receiving instructions from a coach offers verbal feedback. To date, augmented feedback has proven to speed up the learning process and to help acquire a skill (for reviews, see Shea and Wulf, 1999; Magill, 2001; Hodges and Franks, 2002; Hodges and Franks, 2004; Magill and Anderson, 2012; Sigrist et al., 2013).

Currently, Virtual Reality (VR) can be used as a tool to guide and boost motor learning in various settings such as sports and rehabilitation (for reviews, see e.g., Sveistrup, 2004; Holden, 2005; Adamovich et al., 2009; Miles et al., 2012; Neumann et al., 2018): indeed, a virtual environment offers the opportunity to introduce innovative types of augmented feedback that exceed real world opportunities (e.g., Todorov et al., 1997; Chua et al., 2003; Sigrist et al., 2013, 2015). For instance, in the real world the learner can compare her own performance as seen in a mirror to the coach's demonstration of an optimal performance. To do so, the learner must map their own performance to the target performance. This requires some cognitive effort: the learner has to switch between looking at herself in the mirror and looking at the coach, while trying to infer what might be wrong with the movement during its execution. Instead, in VR this effort can be reduced by showing the target performance superimposed on the learner's performance during execution. Here, we developed a VR system for the learning and coaching of full body movements, and provided concurrent visual feedback through a virtual mirror to investigate the influence of superimposing a skilled performance on one's own performance during movement execution.

The impact of augmented visual feedback on motor learning is highly dependent on the content of the feedback provided (Magill and Anderson, 2012). For instance, Shea and Wulf (1999) asked participants to maintain their balance on a stabilometer platform, while receiving concurrent feedback on a screen indicating their deviations from the horizontal, and found this feedback to enhance learning, as measured in a subsequent retention test. From research on observation and modeling (for reviews, see McCullagh et al., 2012; Anderson et al., 2014; Law et al., 2017), the type of model (defined as an example to imitate) shown during practice has proven to be a critical variable for motor learning (Martens et al., 1976; Andrieux and Proteau, 2014). Specifically, it has been shown that watching successful performance promotes motor learning (e.g., Martens et al., 1976), and that mixing successful performance (expert model) and unsuccessful performance (novice model) has proven to be extremely effective for motor learning (Rohbanfard and Proteau, 2011; Andrieux and Proteau, 2013, 2014). For instance, Andrieux and Proteau (2014) found that watching both a novice and an expert model in an alternate fashion favors motor learning as compared to watching either type of model alone. This combination of skilled and unskilled performance can help novices to combine descriptive and prescriptive knowledge of performance and to combine information on movement quality of what is and what should be. Thus, in order to assist a novice in learning a motor skill, providing both information on one's own movement together with a skilled performance during movement execution might be most effective for motor skill acquisition.

To date, several studies have used VR to investigate the influence of observing one's own and/or a skilled performance on subsequent motor performance and motor learning (Todorov et al., 1997; Chua et al., 2003; Burns et al., 2011; Anderson et al., 2013; Covaci et al., 2014; Sigrist et al., 2015; Tang et al., 2015; Hoang et al., 2016). In these studies, the skilled performance has either been visualized as an overlay on top of the participant's movement (e.g., Sigrist et al., 2015) and/or has been visualized on a virtual character next to the participant (e.g., Chua et al., 2003). Sigrist et al. (2015) examined concurrent visual feedback in a VR-based rowing simulator in comparison to different types of multimodal feedback. In their visual feedback condition, the target movement of the oar was visualized as an overlay on top of the participant's oar. Depending on the deviation from the target, the transparency of the target oar changed, and a trace of the participant's trajectory was shown when the error became too large. The authors observed improvements in spatial error as well as in temporal error for all conditions, including unimodal visual feedback, showing that unimodal feedback was as effective as multimodal feedback in their study. While Sigrist and colleagues chose to superimpose the skilled use of a tool (i.e., the oar) on participants' performance, to focus on one body part (i.e., the arm), and to manipulate additional information in the visual feedback condition (i.e., trace visualization, changes in opacity), the mere effect of visual feedback cannot be inferred from this particular study and it remains unclear whether their findings generalize to full body movement without tool use.

With regards to full body movement, Chua et al. (2003) investigated the impact of different visual feedback strategies on Tai Chi performance, including two conditions with concurrent superimposition of a virtual character executing a skilled performance. In their study, however, superimposing the virtual coach on the participant's virtual body did not lead to any effect. Thus, whether superimposing a skilled performance of a full body movement on that of the learner's virtual body during movement execution is beneficial to motor learning is still unclear. To the best of our knowledge, among the few studies that focus on full body movements (e.g., Chua et al., 2003; Burns et al., 2011; Hoang et al., 2016), no systematic investigation of this feedback strategy on motor learning exists that allows the determination of the mere effect of a superimposed skilled performance. Nonetheless, most sports require the execution of full body movements. The coordination of a full body movement with many degrees of freedom is effortful for novices and superimposing a skilled performance on novices' own performance may thus pose additional demands on information processing against the background of limited processing capacities (Guadagnoli and Lee, 2004). On the other hand, especially novices have been shown to profit from concurrent feedback, as they do not yet have a representation of the skill in an early phase of learning (Hodges and Franks, 2002, 2004; Frank et al., 2013, 2018b). Concurrent feedback thus guides them during movement execution (Salmoni et al., 1984; Todorov et al., 1997; Marschall et al., 2007; Sigrist et al., 2013). It is therefore unclear whether novices can profit from watching a successful performance superimposed on their own performance during movement execution, or whether the superimposition of a full body movement provides too much information for a novice, and thus hinders learning.

A related factor that is important in motor learning is the participant's viewing perspective, as it determines which perceptual information can be picked up by the observer for subsequent action execution (Scully and Newell, 1985). For many exercises, the crucial aspects of the movement cannot be well observed from a frontal perspective. For instance, common errors while practicing squats involve wrong weight distribution or bending the back in a wrong way. In a real environment, such as a gym, a person can have a side perspective of the movement from a mirror during movement execution only when turning the head, which would imply a wrong posture for the squat. As opposed to the real world, virtual environments allow for changes in perspective (Salamin et al., 2010; Covaci et al., 2014; Hoang et al., 2016). For instance, while in the real world participants watch themselves in a mirror looking at their own performance from a natural perspective, artificial rotations in VR allow for different perspectives, such as watching oneself from the side whilst facing the mirror. To the best of our knowledge, while some studies investigate different perspectives (e.g., Salamin et al., 2010; Covaci et al., 2014), and even though combinations of different perspectives with overlays exist (e.g., Hoang et al., 2016), there is no investigation of varying perspectives together with full body superimposition of skilled performance.

Apart from motor performance as a valid indicator of motor learning (Schmidt and Lee, 2011), learning can as well be tracked in terms of changes in cognitive representations in motor memory (Frank et al., 2013). According to the cognitive action architecture approach (CAA-A; for an overview, see Schack, 2004; Schack and Mechsner, 2006), motor actions are hierarchically organized across cognitive and motor levels and are represented in memory as well-integrated representational networks. These cognitive representations of motor actions are comprised of basic action concepts (BACs). Similar to the idea of object representations and basic object concepts (e.g., Rosch and Mervis, 1975; Rosch, 1978; Mervis and Rosch, 1981; Hoffmann, 1986, 1990), BACs represent cognitive compilations of movement elements and body postures and their corresponding perceptual effects, being closely tied to the attainment of action goals (e.g., Schack, 2004, 2012). BACs are encoded in long-term memory and guide motor skill execution (Schack and Mechsner, 2006; Land et al., 2013). While experts' representation structures have been shown to be organized in a hierarchy, with groupings of BACs matching well the biomechanical demands of the task, representation structures of novices vary more among individuals, are less hierarchical, and reveal fewer and less functional groupings of BACs (Schack and Mechsner, 2006; Bläsing et al., 2009). Learning, according to the CAA-A, is reflected by modifications in the relations and the groupings of BACs and the respective representation structure, and thus by functional changes in representational networks of complex action in long-term memory (e.g., Schack, 2004; Schack and Ritter, 2013). Together with performance improvements, novices' representations have been shown to become functionally more organized following physical practice (Frank et al., 2013) and mental types of practice, such as motor imagery (Frank et al., 2014, 2018a) and action observation (Frank et al., 2018b). The impact of VR-based augmented feedback on the development of cognitive representations, however, remains to be explored.

**Figure 1:**
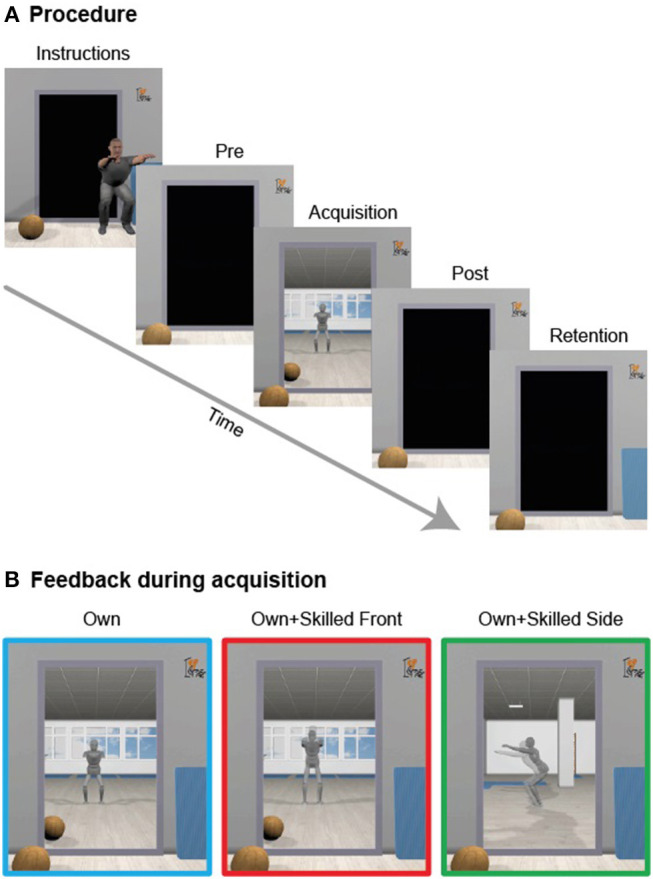
Procedure and conditions. **(A)** The experiment consisted of different phases, and participants were asked to perform squat movements. **(B)** During acquisition, participants were provided with different visual feedback: one group of participants observed only the own avatar. A second group observed the skilled performance superimposed onto the own avatar from a frontal perspective. A third group watched the skilled performance superimposed over their own avatar from a side view.

Accordingly, the purpose of the present study was to investigate the influence of superimposing a skilled performance on one's own performance in a virtual mirror during the execution of a full body movement (here: the bodyweight squat). Specifically, concurrent visual feedback was provided such that participants watched their own performance of a squat in front of a virtual mirror (a situation analogous to watching themselves in a real mirror), or with a superimposed skilled performance, either from the front or from the side (Figure 1B). In particular, we aimed to explore whether the novice participants would tend to spontaneously adjust their movements in order to match them with the correct ones, and whether this would benefit motor learning compared to watching one's own performance alone. This was realized in a state-of-the-art immersive environment, designed for the coaching of motor actions in VR (Waltemate et al., 2015). To this end, we mapped the participant's performance to a virtual avatar and showed this performance in a virtual mirror during the execution of a squat movement. At the same time, we showed the performance of a skilled individual mapped onto a second virtual character, superimposed over the participant's avatar.

We investigated the effectiveness of these different kinds of visual feedback on motor performance of the squat and its cognitive representation in long-term memory. We hypothesized that superimposing a skilled performance would lead to better motor performance and more developed cognitive representations compared to watching one's own performance alone. To this end, we compared the motor performance of the learner with the skilled one along different kinematic and temporal parameters, and we investigated whether the different types of visual feedback would lead to reductions in error performance. Similarly, we compared cognitive representation structures to a reference to examine whether the structures changed toward more functional ones, and whether this change depended on type of visual feedback. Furthermore, we expected an influence of perspective on these two variables. Specifically, the superimposition was, in an additional condition of our experiment, enriched by a rotated perspective in the virtual mirror: participants performing in front of a virtual mirror observed their own movement together with the skilled performance from the side. Such a rotation might offer the advantage of watching body parts that are crucial in the execution of the squat, and that are not visible from a frontal (and natural) point of view. This may allow for an easier error correction. On the other hand, the rotation in perspective might interfere with the performance, and require the participants to perform a mental rotation of the image, which might have a detrimental effect in sensorimotor learning, instead of facilitating it.

To control for participants' subjective experiences with the virtual characters, we furthermore assessed whether participants felt like they owned and they were in control of the virtual character (sense of ownership and sense of agency: Tsakiris et al., 2006; Kilteni et al., 2012, 2015). This was done first to assess whether a possible lack of error reduction during the training in one or more groups, might have been due to a lack of sense of ownership and agency toward the virtual characters, as both are known to affect motor learning tasks (e.g., Alimardani et al., 2016; see also Adamovich et al., 2009). Second, we wanted to explore whether possible differences arising in the motor performance across groups could be associated with differences in body ownership and sense of agency, since the perspective of view is known to affect the sense of ownership (Maselli and Slater, 2013). Finally, we also explored the perceived plausibility and simultaneity of the virtual characters, as they can also affect motor performance, as well as sense of agency and ownership (Waltemate et al., 2016).

## Experiment 1

### Materials and Methods

Three groups of novices (between-subject design) performed bodyweight squats inside a virtual environment while obtaining concurrent visual feedback through a virtual mirror (Figure 1 and the Supplementary Video). We chose the squat as it is a self-paced action of relatively low speed that can be executed while staying in the same place, and as such it is suitable to be executed in a CAVE (for details, see Apparatus). Moreover, technique and movement quality are a key factors when executing the squat (as opposed to a golf putt, for instance, where holing the ball is the primary goal of the action). Although novices can execute the action as a whole, they do differ from more skilled individuals in their technique, and typically show erroneous performance with room left for improvement. For these reasons, the bodyweight squat was considered a suitable action for coaching in VR allowing for the provision of concurrent and superimposed feedback.

Concurrent visual feedback was provided such that participants either watched their own performance of a full body movement in front of a virtual mirror alone (Own), or with a superimposed skilled performance, either from the front (Own+skilledFront) or from the side (Own+skilledSide). We investigated the impact of these different kinds of feedback on motor performance, cognitive representation, and subjective judgments.

### Participants

Thirty-five healthy, naïve participants (21 males, mean age *M* = 26.3, standard deviation *SD* = 4.4) took part in the study. We determined the sample size based on a power analysis, with an expected medium effect size, alpha set at 0.05, and a desired power level of 0.80, as suggested by Cohen (1988). Four further participants were tested, but their data were not included in the analyses due to technical issues during the experimental session. All participants were novices with respect to the squat: they had never attended a professional training of the exercise before, had never trained the squat on a regular basis and did not have any theoretical information on how to execute the squat properly. All participants were taller than 1.6 m and spoke German fluently.

Participants provided written informed consent and got paid 6 euros per hour for their participation. The study was conducted in accordance with the Declaration of Helsinki and had ethical approval from the ethics committee of Bielefeld University.

### Apparatus

Our VR setup consisted of a CAVE (Cruz-Neira et al., 1992) with two projection walls (front and a floor, 3 × 2.3 m for each side), as well as an optical motion capture system. Each of the CAVE walls was operated by two projectors (Projection Design F35 WQ) at a resolution of 2,100 × 1,600 pixels per projector to allow for stereoscopic 3D visualization. The images were updated at 60 Hz. For image separation, we used passive INFITEC filters. The whole graphics environment was controlled by a self-developed custom rendering engine that ran on a single computer equipped with two NVIDIA Quadro K5000 graphics cards. Each of them was connected to the two projectors of one wall of the CAVE.

**Figure 2:**
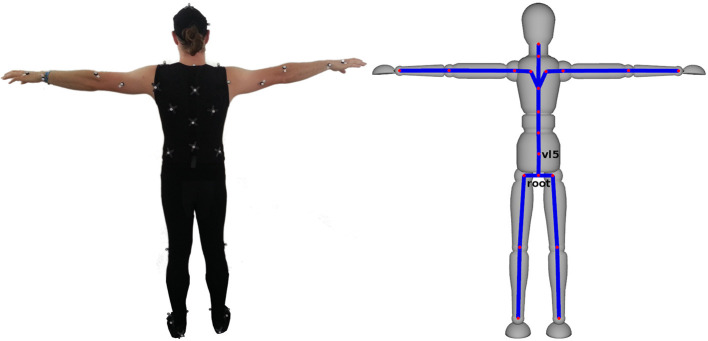
Marker setup and reconstructed skeleton representation. Joints that are specifically used for the kinematic analysis are named.

For motion capturing, we used a passive marker-based outside-in tracking system by OptiTrack. It consisted of ten Prime 13W cameras and was used to track 44 markers for full body motion capture together with six markers attached to the participant's glasses for perspective adaptation. For full body motion capture, participants wore a partly self-designed tight-fitting suit. Parts of the markers were attached to the suit; the others were directly attached to the participants' skin. Our marker setup resulted in the reconstruction of 21 joint rotations (see Figure 2) and joint translations at 120 Hz. To obtain this information, we followed a multi-step calibration procedure. First, the position of the cameras was calibrated. Next, participants wearing the marker suit were calibrated inside the CAVE. Then, the participants were asked to move their arms and legs inside the tracked area and the experimenter compared the actual movement with the movement reconstructed by the motion capture environment. In case of observed reconstruction problems, the marker setup was iteratively refined.

During the experiment, we placed the participants inside a virtual room which was rendered on the walls of the CAVE. Depending on the phase of the experiment, this room was equipped with either a black plane or a virtual mirror in front of the participant (Figure 1A). If the mirror was shown, it reflected the virtual room as well as a virtual avatar of the participant. This avatar had the appearance of a wooden stick figure, with a per-limb scaling according to the participant's limb lengths. The avatar was animated in real time using the information from the motion capture system. The rendering engine ran at around 88 Hz. From evaluations of the system in previous experiments (Waltemate et al., 2015), a latency of around 60 ms was measured for an environment which closely resembled the environment used in this experiment. Moreover, from a previous study involving the same experimental setting and a similar task, in which we investigated the impact of different delays (ranging from 45 to 350 ms) on motor performance, sense of agency, sense of body ownership, and simultaneity perception during the execution of several full body movements, we showed that participants perceived the visual feedback as perfectly simultaneous for delays up to around 75–100 ms, while sense of agency and ownership started to drastically decay at much higher latencies (around 210–300 ms). Thus, based on those previous findings, the latency we had in the present study (i.e., around 60 ms) should guarantee simultaneity perception, sense of agency and ownership (Waltemate et al., 2016).

### Procedure

The experiment consisted of four phases: pre-test, acquisition, post-test, and retention test. Pre-test, acquisition phase, as well as the post-test took place on the first day and lasted ~2 h. The retention test took place on the day after and lasted around 1 h. Participants were assigned to one of three groups: Own (*n* = 12), Own+skilledFront (*n* = 11), Own+skilledSide (*n* = 12) which differed in the content of concurrent visual feedback provided in the acquisition phase (see “Acquisition phase” section and Figure 1). Each group was gender balanced.

#### Pre-test

First, we handed out general information about the overall experiment as well as a consent form. In the next step, participants filled in questionnaires for demographic data and simulator sickness (Kennedy et al., 1993). Then, we equipped participants with 3D glasses and asked them to enter the CAVE and to stand on a marker on the floor of the virtual room. Participants were asked to carefully observe a virtual character performing a skilled squat twice. The skilled squat was a recording of a skilled athlete (8 years of experience in practicing the squat for 2–3 times per week) and was shown from a front view. Participants were asked not to move while watching the prerecorded performance.

Next, participants left the CAVE and performed a split procedure to measure their cognitive representation of the squat in long-term memory (Structural Dimensional Analysis of Mental representations; SDA-M, see Schack, 2012; for details, see Data Analysis).

After completion of the split procedure, participants put on a motion capture suit and were equipped with motion capture markers. Next, we asked participants to perform a single squat and instructed participants to reach approximately the desired depth (around 100 degrees). With this step we aimed at preventing them from performing the movement too deep, which would have put too much strain on their knees. In the next step, we equipped participants again with the 3D glasses and asked them to orient themselves toward the black mirror on the front wall of the CAVE while standing on the marked position on the floor. Again, a virtual character demonstrated the prerecorded skilled squat for two consecutive times, and after that disappeared from the screen. Then, the participants' initial squat performance was recorded. We asked them to perform the movement as similarly as possible in terms of body postures and temporal aspects to the recording of the skilled person they had previously seen. Participants started the recording procedure themselves by performing a T-Pose. Then, they performed ten repetitions of a single squat in two blocks of five repetitions each. Instructions on when to start the squat together with countdowns (from 5 to 0, with 0 representing the go-signal) were presented in textual form on the black mirror in front of the participant.

#### Acquisition Phase

In order to familiarize with the environment, participants were asked to move about freely for 45 s in in the center of the CAVE, while watching their own avatar in the virtual mirror. After familiarization, participants performed 6 blocks of 5 squats each. We asked participants to perform the exercise as similarly as possible to the performance of the skilled person as shown during pre-test. Participants performed the motor task under different concurrent visual feedback conditions (Figure 1B): participants in the Own group observed their own avatar from the front in the mirror during the squat. Those in the Own+skilledFront group observed their own avatar as in the Own group from the front, but together with a second virtual character superimposed on their own. The second character performed the skilled performance as demonstrated before the pre-test. It was scaled in the same way as the participant's avatar and displayed slightly transparent. Participants in the Own+skilledSide group observed the same scene as those in the Own+skilledFront group, but with the mirror image rotated by 90 degrees around the vertical axis. Thus, they saw their own performance as well as the skilled performance from the side. The participants in the Own+skilledFront and Own+skilledSide groups were informed that they were going to observe the movement of the skilled person.

#### Post-test

The procedure in the post-test was the same as in the pre-test for the two blocks of five squats. In addition, questionnaires on simulator sickness and about participants' experience in the virtual environment were filled out (cf. **Table 3**). Participants in the Own+skilledFront and Own+skilledSide groups were asked to answer questions related to the avatar twice, once for their own avatar and once for the skilled avatar. Finally, the experimenter removed the markers and participants pulled off the motion capture suit.

#### Retention-Test

The procedure in the retention-test was the same as in the pre- and post-test. The retention-test took place 1 day after. First, participants put on the marker suit and markers were attached again. We used photos of the participant as well as the calibration data inside the motion capture system from the day before to verify the positioning of the markers. After having performed 10 squats, participants put off the marker suit and performed the split procedure again in order to measure their final cognitive representation of the squat.

### Measures and Data Analysis

#### Motor Performance

**Figure 3:**
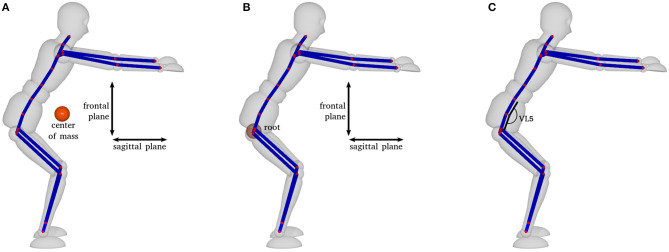
Kinematic variables measured at the deepest point of the movement: **(A)** center of mass (com) based on the centroid of the joint positions, **(B)** position of the hips based on the root joint [both variables on the sagittal plane (back vs. front) and on the frontal plane (up vs. down)], and **(C)** flexion of the back based on the angle of joint vl5.

Motor performance was measured using motion capture data (for details, see Apparatus). Based on these data, we focused on (a) spatial and temporal comparisons of the whole movement with a skilled movement based on Dynamic Time Warping (DTW), (b) a comparison between the motor performance of the participant and that of the skilled athlete, as measured by error performance along several kinematic variables measured at the deepest point of the movement (see Figure 3), and (c) numbers of principal components required to specify participants' movements based on principal component analysis (PCA, see Bishop, 2006; for details on how motor performance measures were calculated, see Supplementary Material). For the kinematic variables at the deepest point, we focused on five measures of the performance error. The first two variables were based on a simplified center of mass (com) that was determined based on the centroid of the joint positions either on the sagittal plane (back vs. front) or on the frontal plane (up vs. down; see Figure 3A). Two further measures were based on the position of the hips (root joint, see Figure 3B), also on the sagittal plane or on the frontal plane. The fifth measure compared the flexion of the back, based on the angle of joint vl5 (see Figure 3C). We chose these five variables as they are, from an applied point of view, critical aspects for the quality of the squat (IFHIAS, 2013). Both the position of the hips and the com relate to weight distribution, while the flexion of the back is a marker of overall posture of the upper body. Specifically, to prevent too much knee strain, it is important to move the hips (and thus the com) backwards while going down, and the flexion of the back is relevant as a straight back protects from back pain. Each of the five variables was calculated in comparison to the skilled performance to estimate the error for each time of measurement.

For each parameter, a repeated-measures analysis of variance (ANOVA) was conducted on performance error with phase (pre-test, acquisition, post-test, retention-test) as within-subject factor and group (Own, Own+skilledFront, Own+skilledSide) as between-subject factor. For all analyses, the level of significance was set at *p* < 0.05. *post hoc* comparisons were run with a Bonferroni correction. In case of sphericity violation, the Greenhouse-Geisser correction for repeated measures was applied. To evaluate a possible effect of the training on the number of the principal components (i.e., on structural changes in the performance), we ran a Friedman test in each group on the number of principal components in the pre-, post-, and retention-test.

#### Cognitive Representation

In order to measure participants' cognitive representations of the squat in long-term memory (for details on the CAA-A, see introduction) by way of psychometric data, Structural Dimensional Analysis of Mental representation (SDA-M; Schack, 2012) was employed. The SDA-M serves to determine relations between basic action concepts (BACs) and to outline the structure of one's cognitive representation. For the specific purpose of the present study, 16 concepts were used (see Table 1), each relating to a particular movement phase: preparation phase (BAC 1–3), going-down/main phase (BAC 4–10), going-up/ attenuation phase (BAC 11–12), or relating to typical error patterns (BAC 13–16). This set of 16 BACs was determined based on experts' and coaches' ratings of a preliminary set of BACs.

**Table 1:** Basic action concepts (BACs) of the squat.

**N^**°**^**	**Basic action concept (BAC)**	**Phase/Errors**
1	Shoulder-width stance	Preparation
2	Toes slightly rotated outwards	
3	Upright posture	
4	Bend legs	Main phase
5	Push bottom backward	
6	Keep upright posture	
7	Knees remain behind toes	
8	Knees remain in same axis as feet and hip joints	
9	Heels remain on the ground	
10	Knee angle 100°	
11	Push hips forward	Attenuation
12	Extend legs	
13	Push knees forward	Error patterns
14	Knees point inwards	
15	Heels leave the ground	
16	Bend upper back	

Specifically, as a first step of the SDA-M, a split procedure was performed in front of a computer: while one BAC was permanently shown on a screen (i.e., anchor position), the remaining concepts were presented one after another in randomized order. For each pair of concepts being displayed, participants were asked to decide whether the two concepts related to one another during movement execution or not, splitting the set of concepts into subsets. Once the participants had finished a list of concepts, another concept took the anchor position and the procedure continued. Once each concept had been compared to the remaining ones, the split procedure was completed.

Based on individual distance scalings between BACs as obtained from the split procedure, a hierarchical cluster analysis (average linkage) was performed to outline the structure of the cognitive representation for each group and each test phase (α = 0.05; d_crit_ = 3.41). An analysis of invariance within- and between-groups served to compare different cluster solutions, and thus to track the change in cognitive representation structures. According to Schack (2012), two cluster solutions are variant, that is significantly different, for λ < 0.68, while two cluster solutions are invariant for λ ≥ 0.68. In addition, the similarity between representation structures and a reference structure reflecting well the different movement phases was examined. For this analysis of similarity, Adjusted Rand Indices (ARI; Santos and Embrechts, 2009) were calculated for each group and time of measurement in comparison to the reference, in order to rank similarity of mean group tree diagrams relative to this reference. Indices between “−1” (cluster solutions are different) and “1” (cluster solutions are the same) mark the degree of similarity. This analysis helped to determine whether the change in cognitive representation structures reflected a functional development toward an expert structure.

While experts' representation structures have been shown to be organized in a hierarchy, with groupings of BACs matching well the biomechanical demands of the task, representation structures of novices vary more among individuals, are less hierarchical, and reveal fewer and less functional groupings of BACs (Schack and Mechsner, 2006; Bläsing et al., 2009). During learning, however, novices' representation structures typically develop toward more hierarchical ones after practice (Frank et al., 2013), and evidence of such development would thus be a marker for functional changes in long-term memory.

#### Subjective Judgments

Questionnaires were used to measure simulator sickness (Kennedy et al., 1993), and the experience during the acquisition phase with regard to sense of agency and ownership toward the own avatar, perceived latency of the avatar, its anatomical plausibility, and two control questions (cf. Table 3). Questions of the second questionnaire were answered on a 7-point Likert scale, ranging from −3 to + 3 (+3 indicated maximum agreement). The items of the latter questionnaire were formulated by adapting questionnaires from previous studies, which investigated sense of ownership and agency (Lenggenhager et al., 2009; Slater et al., 2010; Tsakiris et al., 2010; Maselli and Slater, 2013), as well as simultaneity perception (Waltemate et al., 2016).

To test for the presence of simulator sickness induced by the system, we compared the responses of each item of the simulator sickness questionnaire between the first and the second presentation of the questionnaire using the Wilcoxon Signed rank test. Moreover, for each item and group we calculated the mean differences between post- and pre-test scores and compared those differences across the different groups by means of the Kruskal–Wallis one-way analysis of variance.

For the experience questionnaire (cf. Table 3), we calculated the mean response in each item and group. For questions relating to the participant's avatar, differences across the three groups were tested by means of the Kruskal–Wallis one-way analysis of variance. In case of significant results, *post hoc* comparisons were calculated by means of Wilcoxon rank sum test. For questions relating to the character that was used to display the skilled performance, Wilcoxon Signed rank tests were used for each item of the experience questionnaire to test whether each response significantly differed from zero.

### Results

#### Motor Performance

**Figure 4:**
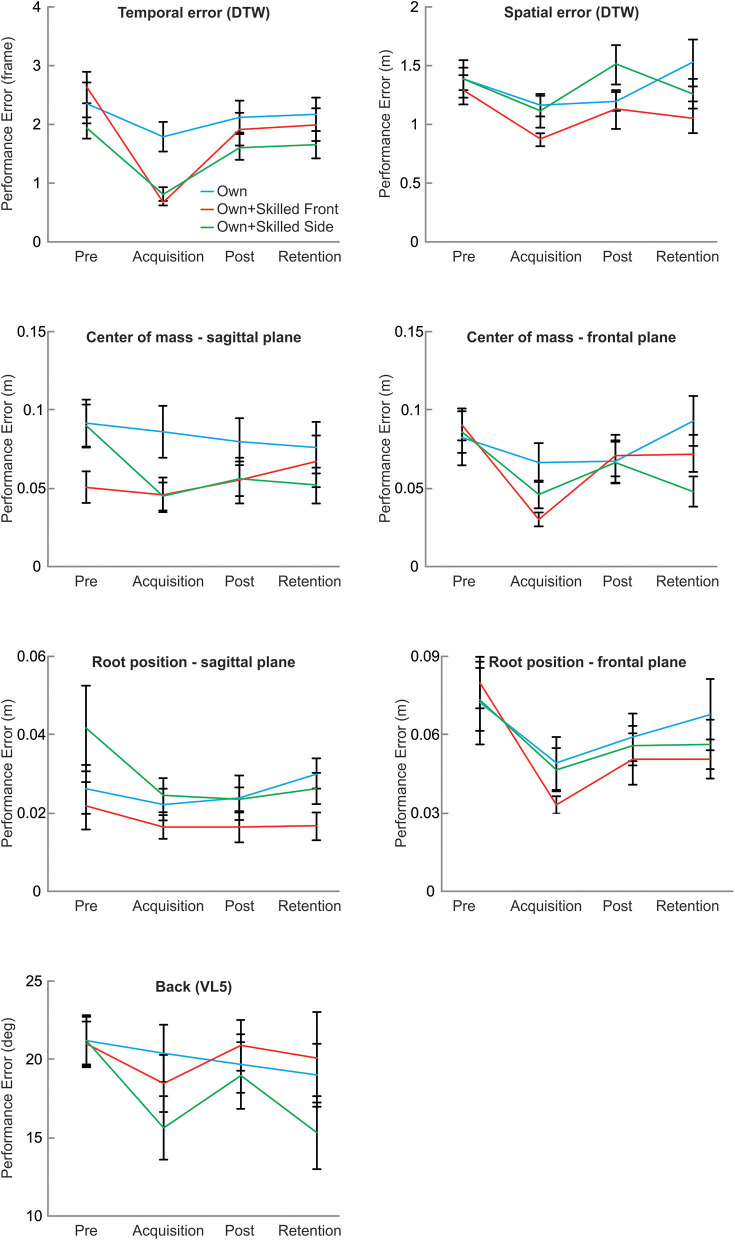
Motor performance results. Each graph shows the effect of the visual feedback provided to the different groups on each parameter used to evaluate motor performance.

Results for motor performance variables are displayed in Figure 4. Means and standard deviations are reported in Table 2. The three groups did not differ in their ability to perform the squat before the beginning of the experimental session, as shown by the lack of significant differences in the pre-test phase across groups in each of the aforementioned parameters (all *p* > 0.80)^1^.

**Table 2:** Descriptive statistics for kinematic variables across pre-test, post-test, acquisition phase, and retention-test for the different feedback groups.

	**Pre-test**	**Acquisition**	**Post-test**	**Retention-test**
	***M*** **±*****SD***	***M*** **±*****SD***	***M*** **±*****SD***	***M*** **±*****SD***
**SPATIAL ERROR**				
Own	1.38 ± 0.56	1.16 ± 0.33	1.19 ± 0.29	1.52 ± 0.70
Own+skilledFront	1.30 ± 0.42	0.87 ± 0.18	1.12 ± 0.55	1.06 ± 0.44
Own+skilledSide	1.38 ± 0.38	1.11 ± 0.54	1.51 ± 0.65	1.26 ± 0.50
**TEMPORAL ERROR**				
Own	2.36 ± 1.19	1.79 ± 0.87	2.13 ± 0.94	2.17 ± 1.00
Own+skilledFront	2.62 ± 0.88	0.66 ± 0.15	1.92 ± 0.93	1.99 ± 0.92
Own+skilledSide	1.93 ± 0.74	0.81 ± 0.44	1.61 ± 0.84	1.65 ± 0.92
**COM—SAGITTAL**				
Own	0.09 ± 0.05	0.09 ± 0.06	0.08 ± 0.05	0.08 ± 0.06
Own+skilledFront	0.05 ± 0.03	0.05 ± 0.04	0.06 ± 0.05	0.07 ± 0.06
Own+skilledSide	0.09 ± 0.05	0.04 ± 0.03	0.06 ± 0.04	0.05 ± 0.04
**COM—FRONTAL**				
Own	0.08 ± 0.06	0.07 ± 0.04	0.07 ± 0.05	0.09 ± 0.06
Own+skilledFront	0.09 ± 0.03	0.03 ± 0.02	0.07 ± 0.04	0.07 ± 0.04
Own+skilledSide	1.03 ± 0.05	0.79 ± 0.03	0.97 ± 0.05	0.97 ± 0.04
**ROOT—SAGITTAL**				
Own	0.03 ± 0.02	0.02 ± 0.01	0.02 ± 0.02	0.03 ± 0.01
Own+skilledFront	0.02 ± 0.02	0.02 ± 0.01	0.02 ± 0.01	0.02 ± 0.01
Own+skilledSide	0.04 ± 0.04	0.02 ± 0.02	0.02 ± 0.01	0.03 ± 0.01
**ROOT—FRONTAL**				
Own	0.07 ± 0.06	0.05 ± 0.04	0.06 ± 0.03	0.07 ± 0.05
Own+skilledFront	0.08 ± 0.03	0.03 ± 0.01	0.05 ± 0.03	0.05 ± 0.03
Own+skilledSide	0.07 ± 0.05	0.05 ± 0.03	0.06 ± 0.03	0.06 ± 0.04
**Vl5**				
Own	21.18 ± 5.66	20.36 ± 6.27	19.72 ± 6.43	19.01 ± 7.04
Own+skilledFront	20.98 ± 4.92	18.46 ± 6.19	20.91 ± 5.46	20.10 ± 9.58
Own+skilledSide	21.17 ± 5.86	15.62 ± 7.98	18.96 ± 8.23	15.30 ± 9.10

*com, center of mass; sagittal plane, back vs. front; frontal plane, up vs. down; temporal error is reported in frames, spatial error, com and Root in meters, and Vl5 in degrees*.

Analyses of variance revealed significant main effects of phase for all variables (spatial error: *F*_3, 96_ = 3.78, *p* = 0.013; temporal error: *F*_3, 96_ = 29.74, *p* < 0.001; the deviation of center of mass (com) in the sagittal plane (*F*_3, 96_ = 5.21, *p* = 0.002) and in the frontal plane (*F*_3, 96_ = 8.99, *p* < 0.001); the deviation root position in the sagittal plane (*F*_3, 96_ = 3.20, *p* = 0.046) and in the frontal plane (*F*_3, 96_ = 8.61, *p* < 0.001); and the deviation of the angle between hips and upper body (*F*_3, 96_ = 4.49, *p* = 0.005).

*Post hoc* comparisons showed that the temporal error, the deviation of the com at the deepest point in the sagittal plane, and the deviation of the root position in the sagittal plane decreased in acquisition, post-test and retention, as compared to the pre-test (all *p* < 0.05). For the deviation of the angle between hips and upper body, the error decreased in acquisition and retention-test as compared to pre-test (all *p* < 0.02). For the deviation of the root position in the sagittal plane, performance error decreased in all groups in acquisition phase and in post-test as compared to pre-test (all *p* < 0.03). For spatial error, a decrease was evident in all groups for acquisition as compared to pre-test (*p* = 0.014). For the com at the deepest point in the frontal plane, the error was smaller in acquisition as compared to pre-test and retention-test (all *p* < 0.02).

The phase by group interaction was significant for temporal error (*F*_6, 96_ = 3.19, *p* = 0.007), and for the deviation of com in the sagittal plane (*F*_6, 96_ = 4.82, *p* < 0.001) as well as in the frontal plane (*F*_6, 96_ = 2.32, *p* = 0.039). It was not significant for spatial error (*F*_6, 96_ = 1.27, *p* = 0.277), the deviation of root position in the sagittal plane (*F*_6, 96_ = 1.31, *p* = 0.259) and in the frontal plane (*F*_6, 96_ = 0.60, *p* = 0.727), and the angle between hips and upper body (*F*_6, 96_ = 1.39, *p* = 0.226).

Temporal error diminished in the Own+skilledFront group and the Own+skilledSide group in the acquisition phase as compared to pre-test (Own+skilledFront: (*p* < 0.001; Own+skilledSide: *p* = 0.003), post-test (Own+skilledFront: *p* < 0.001; Own+skilledSide: *p* = 0.003), and retention-test (Own+skilledFront: *p* < 0.001; Own+skilledSide: *p* = 0.023). For com in the sagittal plane, *post hoc* tests revealed a significant reduction of the performance error in the Own+skilledSide group only, for which motor performance improved in acquisition (*p* < 0.001), post-test (*p* = 0.007), and retention tests (*p* = 0.002), as compared to the pre-test. For com in the frontal plane, error performance decreased in the acquisition phase of the Own+skilledFront group, as compared to pre-test, post-test, and retention-test.

Finally, none of the group effects was significant (spatial error: *F*_2, 32_ = 1.76, *p* = 0.187; temporal error: *F*_2, 32_ = 2.19, *p* = 0.129; com sagittal plane: *F*_2, 32_ = 1.32, *p* = 0.281; com frontal plane: *F*_2, 32_ = 0.62, *p* = 0.541; root position sagittal plane: *F*_2, 32_ = 2.12, *p* = 0.140; root position frontal plane: *F*_2, 32_ = 0.25, *p* = 0.783; hips/upper body angle: *F*_2, 32_ = 0.57, *p* = 0.571).

The Friedman test on the number of principal components showed a significant effect of phase in the Own+skilledFront group only (*X*^2^(2) = 7.72, *p* = 0.02). Pairwise comparison with the Wilcoxon signed-rank test (Bonferroni corrected) revealed that the number of principal components decreased in the Own+skilledFront group in the post-test (*M* = 2.6, *SD* = 1.29) as compared to the pre-test (*M* = 4.36, *SD* = 1.29, *z* = −2.45, *p* = 0.014). The effect was not maintained in the retention-test (*M* = 4, *SD* = 1.7, *z* = −0.6 *p* = 0.55). The Friedman test was not significant in the Own group (*X*^2^(2) = 2.48, *p* = 0.29), where the number of component did not significantly change across pre-test (*M* = 3.50, *SD* = 1.38), post-test (*M* = 3.83, *SD* = 1.59), and retention-test (*M* = 4.2, *SD* = 1.54). Similarly, the number of principal components did not change in the Own+skilledSide group (pre-test: *M* = 3.17, *SD* = 1.4; post-test: *M* = 3.33, *SD* = 1.67; retention-test: *M* = 4.2, *SD* = 1.6; *X*^2^(2) = 1.65, *p* = 0.44).

#### Cognitive Representation

**Figure 5:**
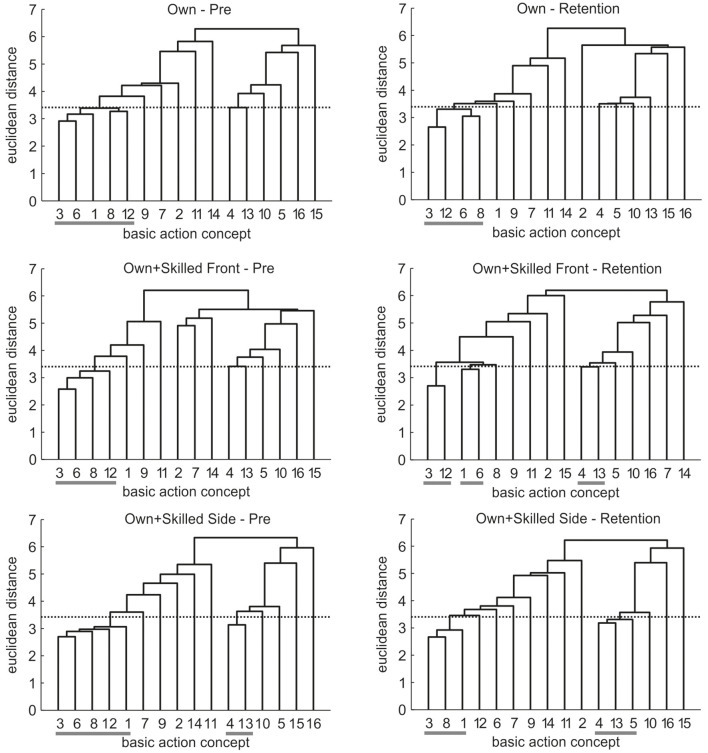
Mean group tree diagrams for Experiment 1 displaying the three visual feedback groups for pre- and retention-test. For each tree diagram, the numbers on the x-axis relate to one particular BAC (for the list of BACs, see Table 1). The numbers on the y-axis display Euclidean distances. The lower the Euclidean distance between BACs, the closer the BACs are. The horizontal dotted line marks the critical value d_crit_ for a given α-level (d_crit_ = 3.41; α = 0.05). Horizontal gray lines on the bottom mark clusters.

Mean group tree diagrams for pre- and retention-test are displayed in Figure 5. For the Own group, the tree diagrams revealed one cluster consisting of several concepts of all three movement phases for both pre-test [1 3 6 8 12] and retention-test [3 6 8 12]. The tree diagrams of the Own+skilledFront group showed a similar cluster for pre-test [3 6 8 12], and three clusters of two concepts each for retention-test [1 6] [3 12] [4 13]. A similar tree diagram was evident for the Own+skilledSide group at pre-test [1 3 6 8 12] [4 13], but at retention-test it revealed more structured clusters [1 3 8] [4 5 13]. Thus, while for the Own and the Own+skilledFront groups, concepts of different movement phases were grouped together after practice, distinct groupings corresponding to distinct movement phases became evident for the Own+skilledSide group.

Analyses of invariance revealed variance across times of measurement for two of the three groups. Specifically, the cluster solutions across time were variant for the Own+skilledFront group (λ = 0.34) and the Own+skilledSide group (λ = 0.63), but not for the Own group (λ = 0.95). This shows that the overall structure of cluster solutions changed over time for the conditions in which participants watched their own avatar together with that of the skilled athlete during movement execution, but not for the condition in which participants watched their own avatar only. Furthermore, adjusted rand indices indicated increasing similarity to the reference for the Own+skilledSide group from pre-test (ARI = −0.05) to retention-test (ARI = 0.03), emphasizing that the mean tree diagram of the group watching the own avatar together with a skilled performance from a 90° rotated perspective revealed a more functional structure after the intervention. In contrast, similarity for the Own group remained stable from pre-test (ARI = −0.03) to retention-test (ARI = −0.03) and decreased slightly for the Own+skilledFront from pre-test (ARI = −0.03) to retention-test (ARI = −0.05).

From these group comparisons, novices' representations changed during learning when watching their own avatar together with that of a skilled person, but not when watching their own avatar only. Particularly, most functional representation structures were evident after having watched their own avatar together with a superimposed skilled performance from the side.

#### Subjective Judgments

Participants did not show simulator sickness after taking part in the experiment (all tested items were not significant, the smallest *p*-value being *p* = 0.37). Moreover, the three groups did not differ in their post-pre-values in any item (all *p*-values being *p* > 0.1). Results of the experience questionnaire are summarized in Table 3. The three groups had analogous sense of agency, ownership, perceived latency, and plausibility toward their own avatar, as shown by the lack of significant differences across groups in all items of the experience questionnaire (all *p* > 0.11). Similarly, the Own+skilledFront group and the Own+skilledSide did not differ in any item relating to the virtual character that was used to display the skilled performance (Wilcoxon rank sum test, all *p* > 0.48). Overall, participants in all groups rated the movements of both virtual characters (own and skilled) as plausible. This indicates that possible tracking errors induced due to the marker suit did not negatively affect the participants' experience. Moreover, they reported a high sense of agency toward their own avatar, and a low latency in the movements of their own avatar, as shown by the Wilcoxon Signed rank test against zero in each item (see Table 3). On the contrary, they neither reported sense of agency, ownership, nor a low latency with respect to their own movement toward the skilled character, as shown by negative values significantly differing from zero (see Table 3).

**Table 3:** Experience questionnaire.

**Questionnaire item**	**Own avatar**			**Skilled avatar**	
	**Own**	**Own+SkilledFront**	**Own+SkilledSide**	**Own+SkilledFront**	**Own+SkilledSide**
The avatar's movements were caused by mine (Agency)	2.8, 0.11^***^	2.9, 0.09^***^	2.4, 0.23^**^	−1.6, 0.62^**^	−1.1, 0.66^**^
I felt like the avatar was my own body (Ownership)	0.3, 0.59	0.8, 0.46	0.2, 0.61	−2.3, 0.45^***^	−1.6, 0.6^**^
The avatar moved as soon as I moved (Latency)	2.4, 0.23^***^	2.4, 0.28^**^	2, 0.39	−1.7, 0.56^**^	−1, 0.8^*^
The movement of the avatar seemed plausible (Plausibility)	2, 0.33^**^	2.2, 0.23^***^	1.6, 0.51^*^	2.1, 0.28^**^	1.7, 0.36^**^
I felt as if I had more than one body (Control quest 1)	−2.6, 0.34^***^	−2.3, 0.33^**^	−2.1, 0.4^**^	−2.3, 0.43^***^	−2.3, 0.35^***^
I felt as if the virtual avatar would move to me (Control quest 2)	−2.7, 0.18^***^	−1.7, 0.59^*^	−2.2, 0.32^***^	−2.2, 0.44^***^	−2.4, 0.19^***^

*Mean and standard deviation in the questionnaire items investigating participants' experience toward the virtual characters (own avatar and the character used to display the skilled performance) in the three groups are reported. The scale ranged from −3 to + 3 (+3 indicated maximum agreement). Asterisks indicate items significantly different from 0*.

## Experiment 2

Although we found an advantage for the Own+skilledFront and Own+skilledSide groups over the Own group in Experiment 1, we observed a significant error reduction in the Own group as well for most kinematics parameters. This might have happened for at least two reasons: participants might have improved their motor performance by directly comparing their own performance, as observed in the virtual mirror, to the instructions watched and memorized at the beginning of the experimental session (i.e., the virtual character performing a skilled squat twice). As an alternative explanation, it could have been the case that merely performing the squat movements several times while thinking at the target performance, irrespective of any visual feedback, might have led to an improvement. In the latter case, it would have been enough for participants to rely on proprioception and compare the feel of the performance to the memorized instruction. If this was true, the visual feedback would not add any further benefit to the improvement: in that case, one would expect that already performing several consecutive squat movements without visual feedback would lead to an error reduction comparable to that observed in the Own group.

To rule out the possibility that some of the improvements observed in Experiment 1 were due to the mere fact of performing the squat movements—irrespective of the type of training received in the acquisition phase—we ran a control experiment. In Experiment 2, participants were presented with a black mirror instead of the virtual mirror during the acquisition phase. If performing repetitive squats without any visual feedback was enough to induce improvements in the motor and cognitive performances in the present experimental design, we expected to find differences in acquisition, post-test and/or retention-test, as compared to the pre-test.

### Participants

Twelve naïve participants (3 males, mean age *M* = 27.33, standard deviation *SD* = 6.6) took part in the study. Participants' selection criteria were the same as in Experiment 1. None of the participants of Experiment 1 took part in Experiment 2. The sample number was chosen based on a power analysis, with an expected medium effect size, alpha set at 0.05, and a desired power level of 0.80, as suggested by Cohen (1988).

### Task and Procedure

The task and the procedure were the same as in Experiment 1. The main difference was that participants executed the squats during the acquisition phase in front of the same black mirror they saw during pre-test, acquisition, post-, and retention-test. Moreover, given that participants were not presented with any virtual character (i.e., the virtual mirror was black), participants did not fill in the questionnaire presented in Experiment 1 on experiences with the virtual characters. Only the questionnaire on motion sickness was filled in, and participants did not show any sign of simulator sickness after taking part in the experiment (all *p* > 0.06).

### Results

#### Motor Performance

For each parameter, a repeated-measures analysis of variance (ANOVA) was conducted with phase (pre-test, acquisition, post-test, retention-test) as within-subject factor^2^. The analyses revealed a significant main effect of phase only for the back (vl5) (*F*_3, 96_ = 5.62, *p* = 0.003). *Post hoc* comparisons revealed a decrease in performance error in the retention-test (*M* = 12.83, *SD* = 1.85) as compared to the pre-test (*M* = 17.87, *SD* = 2.87, *p* = 0.013), and the post-test (*M* = 18.36, *SD* = 2.58, *p* = 0.016). In all other parameters, the main effect of phase was not significant (all *ps* > 0.16), showing that—for most of the tested parameters—the mere execution of squat movements in the absence of visual feedback was not enough to induce improvements in motor performance. Moreover, we ran a Friedman test on the number of principal components in the pre-test, post-test, and retention-test. Results showed a significant increase in the number of principal components (*X*^2^(2) = 8.71, *p* = 0.013) in the post-test (*M* = 8.08, *SD* = 3.6) as compared to both the pre-test (*M* = 3.92, *SD* = 1.2; *z* = −2.55, *p* = 0.01) and the retention-test *M* = 4.42, *SD* = 1.44; *z* = −2.68, *p* = 0.007).

#### Cognitive Representation

The mean group tree diagram for pre-test revealed two clusters, one cluster pertaining to both preparation and main phase [1 3 6 8] and one cluster pertaining to the main phase [4 5]. For retention-test, the diagram was composed of two clusters, one involving concepts of two movement phases [3 6], and one involving an error pattern [4 13]. Analyses of invariance revealed variance across times of measurement (λ = 0.56), indicating that the overall structure of cluster solutions changed over time. However, adjusted rand indices displayed decreasing similarity to the reference from pre-test (ARI = 0.07) to retention-test (ARI = −0.03), indicating that the mean tree diagram changed to a more dysfunctional structure after the intervention.

## Discussion

In the present study, we used a state-of-the-art VR system for the coaching of full body motor actions (Waltemate et al., 2015; de Kok et al., 2017) to provide concurrent visual feedback during the learning of a bodyweight squat. In Experiment 1, we compared the effectiveness of three different types of visual feedback in the acquisition of a proper squat technique: the participant's avatar during the execution of squat trials was presented either alone (Own) or together with the superimposed character used to display a skilled performance, either from the front view (Own+skilledFront) or the side view (Own+skilledSide). Findings from this experiment showed an advantage of the groups observing two avatars, that is their own performance together with the skilled performance over the view of their own performance alone. In Experiment 2, which investigated squat acquisition without any visual feedback, we found a slight tendency of motor performance and cognitive representation to get even worse.

In Experiment 1, participants tended to adapt to the temporal aspects and the depth of the skilled movement. In particular, during the acquisition phase, the Own+skilledFront and the Own+skilledSide groups similarly adapted the timing of their performances to the skilled one. Regarding the center of mass at the deepest point, the Own+skilledFront group reduced the motor error for height during the acquisition phase, indicating an advantage of observing both virtual characters from a front view for correctly estimating how deep participants should go to perform a correct squat. For the center of mass at the deepest point on the sagittal plane, we found an advantage for the Own+skilledSide group in the acquisition phase over the other groups, which was maintained in the retention phase. This indicates that if participants are presented with a side view of the two virtual characters, they can correctly learn how they should adjust their squat along the back-front axis. Thus, we observed changes in motor performance for aspects that could be perceived by the observer according to their particular viewing perspective (Scully and Newell, 1985). Given that we controlled for participants' perceived experiences in the virtual environment, it is unlikely that the differences we found across groups resulted from differences in the way the avatars were perceived across groups. Specifically, according to participants' ratings, participants in all groups perceived avatar's movements as similarly plausible, having a very low latency, and inducing a similar sense of agency and ownership.

Performance error already decreased in participants watching only their avatar—even without a superimposed skilled performance—as compared to the absence of visual feedback. Indeed, performance error decreased for the spatial comparison of participant's movement to the skilled movement and in the positioning of the hips at the deepest point in all feedback groups. This improvement in motor performance was due to the visual feedback (i.e., the observation of the own avatar), and not to the mere repetition of the same movement several times. In fact, practicing the squat without any visual feedback, as realized in Experiment 2, did not significantly improve in their overall motor performance. The participants who performed the squat movement in front of a black mirror and in the absence of direct feedback reduced their error only for one single parameter (vl5; flexion of the lower back), similar to the other groups. Observing their own avatar could be already beneficial to motor learning because participants could directly compare their own performance, as observed in the virtual mirror, to the memorized skilled performance observed during the initial instructions.

Concerning the PCA analysis, we observed a reduction of the principal components at the end of the training for the participants in the Own+skilledFront group. Participants in the other feedback groups did not show any change in the PCA analysis before and after the training. Instead, and opposed to the Own+skilledFront group, executing the task in the absence of any direct feedback increased the number of the principal components, indicating that performance got even worse.

Similar to motor performance, the advantage of providing the avatar of the learner together with that of a skilled athlete is also noticeable in participants' cognitive representation of the squat, as analyzed with the SDA-M (Schack, 2012). Participants in the Own+Skilled groups revealed changes in cognitive representations of the squat in the retention-test. In particular, those who observed the two virtual characters from a side view developed a more structured cognitive representation, which became more similar to that of an expert over the course of practice, and which lasted beyond the training session. Instead, participants who watched their avatar only did not show any change in their cognitive structure. Similarly, and together with an increase in motor performance, previous studies have shown that representation structures develop toward more elaborate ones as a result of practice by execution (Frank et al., 2013) as well as mental types of practice such as observation (Frank et al., 2018b) or imagery (Frank et al., 2014). Moreover, the finding that representations of those who performed the task in the absence of any visual feedback changed toward a more dysfunctional structure indicates that the absence of visual guidance might even lead to a deterioration of the cognitive representation. This together with the PCA findings suggests that performing movements without visual feedback might even be detrimental, as participants in the present study got worse both with regards to the functional groupings of action concepts in their cognitive representation in long-term memory as well as in the number of principal components constituting the overall movement. This is in line with the notion that changes on cognitive levels of action organization are linked to changes on the motor level. For instance, using a spatio-temporal kinematic decomposition of movement together with SDA-M for the full swing in expert golfers, Land et al. (2013) found a close link between movement kinematics and the structure of golfers' cognitive representation of the swing.

While our study shows that observing the participant's own avatar together with the superimposed skilled performance displayed on a second virtual character while practicing a full body movement can improve motor performance, previous studies that focus on complex full body movements (i.e., that are not restricted to one single body part, and not related to the use of tools) did not observe this effect. For instance, Chua et al. (2003) examined the effectiveness of a VR training for Tai Chi, which is a sport that—similarly to a squat—requires the execution of slow full body movements. Performances of a skilled athlete who performed the to-be-learned motor action were shown together with the avatar of the participant. The authors tested several feedback conditions, but did not find any feedback-specific improvements. Such lack of improvement, in contrast to our results, might well be explained with the higher end-to-end latency (around 170 ms) in the setup used by Chua et al. (2003) at that time. Indeed, when participants are presented with concurrent feedback of their movements (e.g., when observing their own virtual avatar), a high end-to-end latency might have affected the perceived temporal coherence of the scene, inducing a break-down in sense of agency and sense of ownership toward the virtual avatar and affecting motor performance (Franck et al., 2001; Longo and Haggard, 2009; Jörg et al., 2012; Imaizumi and Asai, 2015). Instead, the setup that we used in the present study had a low end-to-end latency (around 60 ms, see Waltemate et al., 2015), which might have led, in contrast to Chua et al. (2003), to improvements in motor performance. In a previous study, we asked participants to perform a series of full body movements, and we presented them with their own virtual avatar, whose performance was delayed between 45 and 350 ms (Waltemate et al., 2016). Using a similar set-up as in the present study, Waltemate et al. (2016) showed that awareness for delays significantly increases for an end-to-end latency above 75 ms. Furthermore, perceptual aspects such as sense of agency and ownership were affected for latencies above 125 ms. Most importantly, latencies above 75 ms led to a gradual decay in motor performance (Waltemate et al., 2016). The latency in the setup discussed in Chua et al. (2003) presents an end-to-end latency that according to our previous results would be enough to affect simultaneity perception, motor performance, sense of agency, and ownership (Waltemate et al., 2016). Therefore, the concurrent feedback (i.e., participant's own avatar) would be perceived as less simultaneous to the participants' movement as compared to our setup, and motor performance would drop with increasing delay (see discussion on action-observation network below)^3^.

Similarly, Burns et al. (2011) investigated learning in karate by comparing a “traditional group” (in which a coach gave oral explanations and some practical examples of the movements), a group observing a video of a coach performing a prerecorded example, and a virtual character showing an example of the gestures. The results of this related study showed no significant performance differences after training in the three groups. In our study, however, we showed that a VR environment providing low-latency visual feedback of the learner's avatar together with that of a skilled athlete improves motor performance. In comparison to the conditions in which participants performed the task in the absence of visual feedback or just observing themselves in a virtual mirror, novices can reduce motor error by directly comparing their performance to the target one. Directly comparing one's own to a skilled performance is a clear advantage as compared to what would happen in real training environments, in which learners are provided with instructions and visual examples by the coach, and subsequently have to repeat what they just observed in front of a mirror (or even in the absence of it). This process implies cognitive load: for instance, the learners have to retrieve the relevant information provided by the coach from memory. Moreover, this process is further complicated by the fact that a novice, who by definition has no experience with the to-be-learnt sport, does not know which the most common errors are, and to which body parts he/she should pay more attention in order to avoid such errors. Having the opportunity to directly compare the own avatar to that of a skilled athlete offers an advantage that would not be possible in a real environment. Furthermore, the possibility of showing the two virtual characters from different points of view during movement execution (e.g., from the front, or from the side, which would not be possible in a real environment) provides an additional gain. For instance, participants who observed the virtual characters from a side view were able not only to correct their motor performance (which is completely visible only from the side view) during the training, but also showed improved performance the day after in the retention phase, which indicates motor learning (i.e., the ability to maintain improvements over a period of time and without receiving further feedback; Schmidt and Lee, 2011; Kantak and Winstein, 2012).

A possible suggestion regarding which mechanisms might be responsible for the observed improvement in motor performance comes from the literature on the action-observation network. Previous evidence has shown that action observation, either alone or in combination with the simultaneous execution of a motor task affects behavior and facilitates the acquisition of motor skills (for a review, see Ossmy and Mukamel, 2018). For instance, observing an action while performing a motor task can interfere with or facilitate the ongoing action execution (e.g., Brass et al., 2000; Kilner et al., 2003). Based on neurophysiological and behavioral studies in both humans and animals, it has been suggested that this happens through shared neural representations between action perception and execution (Rizzolatti and Sinigaglia, 2016). In particular, when people observe an action performed by another individual, their motor system internally simulates it, through a matching mechanism that automatically maps the observed action onto the observer's motor repertoire (e.g., Rizzolatti et al., 2001).

Such shared representations, mainly involving frontal and parietal brain areas, not only can induce an interference between perceived and executed movements (as when executing a movement while observing a different one involving the same effector; Kilner et al., 2003), but can also facilitate motor learning, by means of training that combines simultaneous action execution and observation. Nowadays, the use of VR or manipulated videos allows for online manipulation of the visual feedback as it relates to the learner's actual movement execution. For instance, in a recent neuroimaging study, participants were trained in a finger sequence task with their right hand in the presence or absence of different kinds of visual feedback, and performance gain was tested on the left, un-practiced hand after training (Ossmy and Mukamel, 2016). Overall, visual feedback was beneficial to motor learning in the immobile left hand, especially when participants received feedback as if the left immobile hand was the one performing the movement (i.e., when the movements of their right hand controlled the movement of a left virtual hand). Moreover, left hand performance gain correlated with the neural activity during training (Ossmy and Mukamel, 2016). Thus, altering online visual feedback (for instance in terms of involved effector, its size, or movement pace) can boost motor performance and modulate cortical activity (Senna et al., 2015; Ossmy and Mukamel, 2016, 2017a,b). Similarly, in our study we found that online concurrent visual feedback improved performance. Such improvement was even greater when adding a superimposed skilled performance, possibly through the contribution of the action-perception network. The fact that manipulating online feedback in VR leads to greater motor skill acquisition can be also successfully used in rehabilitative settings, where the alteration of online visual feedback in VR has been proven successful in the rehabilitation of stroke patients with motor impairments (e.g., Kang et al., 2012).

Overall, in the present study, participants only showed little learning. Even if they tended to reduce the error in performance during the training, they tended not to preserve the improvement the day after. Only the participants who observed the virtual characters from a side view maintained their performance advantage with respect to the center of mass at the deepest point (sagittal plane: back vs. front) over the retention period. For the other parameters, the improvement in performance was not significantly maintained the day after. This might be mainly due to the fact that we used concurrent feedback during task execution, which is particularly effective for novices (Sigrist et al., 2013), but often leads to a dependency on the feedback (Schmidt et al., 1989; Winstein and Schmidt, 1990; Schmidt, 1991).

A potential limitation in the present study was that we did not investigate the effect of watching a skilled performance only. The comparison of watching a skilled performance to watching a skilled performance superimposed on one's own performance would rule out the possibility that watching the skilled performance only would already lead to similar improvements as observed in the present study. While this seems unlikely, given the well-known advantage of mixing skilled and novice performance in an alternate fashion (e.g., Andrieux and Proteau, 2014), further research is needed to clarify this point. Similarly, it would be interesting to compare the superimposition to showing a skilled performance next to the participant's performance during training (e.g., Chua et al., 2003, but with a lower end-to-end latency comparable to the latency of around 60 ms as used in the present study), in order to determine which of the comparisons is more beneficial for motor learning. Furthermore, while in the present study perspective was a secondary factor to investigate, future studies may fully explore the effect of perspective on watching one's own performance, either alone or together with a superimposition. Finally, future studies should investigate other tasks (e.g., manual action, tool use) than the one used in the present study (i.e., full body movement) to examine whether and how our findings generalize to different types of tasks. Going beyond the scope of the present study, in which we compared different types of augmented feedback in VR, future studies should as well compare this type of VR-based feedback to feedback as provided during real-world coaching as this will shed further light on the effectiveness of feedback provided by technology-based coaching systems as opposed to real human coaches.

To conclude, the present study links observational practice to VR-based concurrent visual feedback and provides insights into new types of augmented feedback during movement execution for the coaching of motor actions in VR. Specifically, this study was the first to show that observing the participant's own avatar together with superimposed skilled performance displayed on a second virtual character while practicing a full body movement can improve motor performance and cognitive representation in memory.

## Ethics Statement

The study was conducted in accordance with the Declaration of Helsinki, and was approved by the Ethics Committee of Bielefeld University. All participants gave written informed consent.

## Author Contributions

FH, CF, IR, ME, TS, and MB: conception and design of study and drafting the manuscript. FH, CF, and IS: data collection and analysis and interpretation of data.

### Conflict of Interest Statement

The authors declare that the research was conducted in the absence of any commercial or financial relationships that could be construed as a potential conflict of interest.

## Footnotes

^1^In addition to balancing gender across groups, we ran an analysis of variance (ANOVA) for each parameter in experiment 1 with the factors phase (pre, acquisition, post, retention) and gender (females, males) in ordert to check whether gender had an influence. The main effect of gender and the phase by gender interaction were never significant in all tested parameters. Thus, gender did not affect the results.

^2^To check whether gender had an influence, we compared the mean performance error and 95% confidence intervals in each phase between males (*n* = 3) and females (*n* = 9). For each parameter and phase, the 95% confidence intervals were always overlapping between females and males, indicating that gender did not affect the results.

^3^To increase replicability of our experiment, according to Waltemate et al. (2015), we report crucial factors, such as framerate of the render engine, latency, and specifications of the hardware. The Supplementary Video helps to further understand the apparatus. As most VR setups differ in single aspects from each other, such parameters must be taken into account when replicating the experiment and when comparing results to related approaches. We would expect similar results for systems that reach a comparable performance in terms of the requirements described in Waltemate et al. (2015).

## References

[B1] Adamovich S. V. Fluet G. G. Tunik E. Merians A. S. (2009). Sensorimotor training in virtual reality: a review. NeuroRehabilitation 25, 29–44. 10.3233/NRE-2009-0497 19713617 PMC2819065

[B2] Alimardani M. Nishio S. Ishiguro H. (2016). The importance of visual feedback design in BCIs; from embodiment to motor imagery learning. PLoS ONE 11:e0161945. 10.1371/journal.pone.0161945 27598310 PMC5012560

[B3] Anderson D. I. Rymal A. M. Ste-Marie D. M. (2014). Modeling and feedback, in Routledge Companionto Sport and Exercise Psychology, eds Papaioannou A. G. Hackfort D (Hove: Routledge), 272–288.

[B4] Anderson F. Grossman T. Matejka J. Fitzmaurice G. (2013). YouMove: enhancing movement training with an augmented reality mirror, in Proceedings of the 26th annual ACM Symposium on User Interface Software and Technology (St. Andrews, UK: ACM), 311–320. 10.1145/2501988.2502045

[B5] Andrieux M. Proteau L. (2013). Observation learning of a motor task: who and when? Exp. Brain Res. 229, 125–137. 10.1007/s00221-013-3598-x 23748693

[B6] Andrieux M. Proteau L. (2014). Mixed observation favors motor learning through better estimation of the model's performance. Exp. Brain Res. 232, 3121–3132. 10.1007/s00221-014-4000-3 24897947

[B7] Bishop C. M. (2006). Pattern Recognition and Machine Learning. New York, NY: Springer Scienceand Business Media.

[B8] Bläsing B. Tenenbaum G. Schack T. (2009). The cognitive structure of movements in classical dance. Psychol. Sport Exerc. 10:350e360. 10.1016/j.psychsport.2008.10.001

[B9] Brass M. Bekkering H. Wohlschlager A. Prinz W. (2000). Compatibility between observed and executed finger movements: comparing symbolic, spatial, and imitative cues. Brain Cogn. 44, 124–143. 10.1006/brcg.2000.1225 11041986

[B10] Burns A. M. Kulpa R. Durny A. Spanlang B. Slater M. Multon F. (2011). Using virtual humans and computer animations to learn complex motor skills: a case study in karate. Skills 2011, 1–4. 10.1051/bioconf/20110100012

[B11] Chua P. T. Crivella R. Daly B. Hu N. Schaaf R. Ventura D. (2003). Training for physical tasks in virtual environments: Tai Chi, in Proceedings of IEEE Virtual Reality (Los Angeles, CA: IEEE), 87–94.

[B12] Cohen J. (1988). Statistical Power Analysis for the Behavioral Sciences. Hillsdale, NJ: Erlbaum Associates.

[B13] Covaci A. Olivier A. H. Multon F. (2014). Third person view and guidance for more natural motor behaviour in immersive basketball playing, in Proceedings of the 20th ACM Symposium on Virtual Reality Software and Technology (Edinburgh, UK: ACM), 55–64. 10.1145/2671015.2671023

[B14] Cruz-Neira C. Sandin D. J. DeFanti T. A. Kenyon R. V. Hart J. C. (1992). The CAVE: audio visual experience automatic virtual environment. Commun. ACM 35, 64–73. 10.1145/129888.129892

[B15] de Kok I. Hülsmann F. Waltemate T. Frank C. Hough J. Pfeiffer T. (2017). The intelligent coaching space: a demonstration, in Lecture Notes in Computer Science: Vol 10498. *Intelligent Virtual Agents: 17th International Conference on Intelligent Virtual Agents from August 27th to 30th in Stockholm*, eds Beskow J. Peters C. Castellano G. O'Sullivan C. Leite I. Kopp S. Sweden (Cham: Springer, 105–108.

[B16] Franck N. Farrer C. Georgieff N. Marie-Cardine M. Dalry J. D'amato T.. (2001). Defective recognition of one's own actions in patients with schizophrenia. Am. J. Psychiatry 158, 454–459. 10.1176/appi.ajp.158.3.454 11229988

[B17] Frank C. Kim T. Schack T. (2018b). Observational practice promotes action-related order-formation in long-term memory: investigating action observation and the development of cognitive representation in complex action. J. Motor Learn. Dev. 6, 53–72. 10.1123/jmld.2017-0007

[B18] Frank C. Land W. M. Popp C. Schack T. (2014). Mental representation and mental practice: experimental investigation on the functional links between motor memory and motor imagery. PLoS ONE 9:e95175. 10.1371/journal.pone.0095175 24743576 PMC3990621

[B19] Frank C. Land W. M. Schack T. (2013). Mental representation and learning: the influence of practice on the development of mental representation structure in complex action. Psychol. Sport Exerc. 14, 353–361. 10.1016/j.psychsport.2012.12.001

[B20] Frank C. Linstromberg G.-L. Hennig L. Heinen T. Schack T. (2018a). Team action imagery: Imagery of game situations and required team actions promotes a functional structure in players' representations of team-level tactics. J. Sport and Exer. Psychol. 40, 20–30. 10.1123/jsep.2017-0088 29565235

[B21] Guadagnoli M. A. Lee T. D. (2004). Challenge point: a framework for conceptualizing the effects of various practice conditions in motor learning. J. Motor Behav. 36, 212–224. 10.3200/JMBR.36.2.212-224 15130871

[B22] Hoang T. N. Reinoso M. Vetere F. Tanin E. (2016). One body: remote posture guidance system using first person view in virtual environment, in Proceedings of the 9th Nordic Conference on Human-Computer Interaction, ACM, 1–10. 10.1145/2971485.2971521

[B23] Hodges N. Franks I. (2004). The nature of feedback, in Notational Analysis of Sport: Systems for Better Coaching and Performance in Sport, eds Franks I. M. Hughes M (London: Routledge, 17–39.

[B24] Hodges N. J. Franks I. M. (2002). Modelling coaching practice: the role of instruction and demonstration. J. Sports Sci. 20, 793–811. 10.1080/026404102320675648 12363296

[B25] Hoffmann J. (1986). Die Welt der Begriffe [The World of Concepts]. Berlin: Verlag der Wissenschaften.

[B26] Hoffmann J. (1990). Über die Integration von Wissen in die Verhaltenssteuerung [On the integration of knowledge into the control of behavior]. Schweizerische Zeitschr. Psychol. 49, 250–265.

[B27] Holden M. K. (2005). Virtual environments for motor rehabilitation: Review. CyberPsychology & Behavior 8, 187–211. 10.1089/cpb.2005.8.187 15971970

[B28] IFHIAS International Fitness and Health Institute of Applied Sciences (2013). Die Tiefkniebeuge [The Deep Squat]. Wissenschaftliche Informationen [Scientific information] (based on an IRON SYSTEMTM Athletic Week Workshop/ Talk by Dirk Büsch).

[B29] Imaizumi S. Asai T. (2015). Dissociation of agency and body ownership following visuomotor temporal recalibration. Front. Integr. Neurosci. 9:35. 10.3389/fnint.2015.00035 25999826 PMC4423341

[B30] Jörg S. Normoyle A. Safonova A. (2012). How responsiveness affects players' perception in digital games, in Proceedings of the ACM Symposium on Applied Perception (Santa Monica, CA), 33–38. 10.1145/2338676.2338683

[B31] Kang Y. J. Park H. K. Kim H. J. Lim T. Ku J. Cho S.. (2012). Upper extremity rehabilitation of stroke: facilitation of corticospinal excitability using virtual mirror paradigm. J. Neuro Eng. Rehabil. 9:71. 10.1186/1743-0003-9-71 23035951 PMC3543207

[B32] Kantak S. S. Winstein C. J. (2012). Learning-performance distinction and memory processes for motor skills: a focused review and perspective. Behav. Brain Res. 228, 219–231. 10.1016/j.bbr.2011.11.028 22142953

[B33] Kennedy R. S. Lane N. E. Berbaum K. S. Lilienthal M. G. (1993). Simulator sickness questionnaire: an enhanced method for quantifying simulator sickness. Int. J. Aviation Psychol. 3, 203–220. 10.1207/s15327108ijap0303_3

[B34] Kilner J. M. Paulignan Y. Blakemore S. J. (2003). An interference effect of observed biological movement on action. Curr. Biol. 13, 522–525. 10.1016/S0960-9822(03)00165-9 12646137

[B35] Kilteni K. Groten R. Slater M. (2012). The sense of embodiment in virtual reality. Presence Teleoperators Virtual Environ. 21, 373–387. 10.1162/PRES_a_00124

[B36] Kilteni K. Maselli A. Kording K. P. Slater M. (2015). Over my fake body: body ownership illusions for studying the multisensory basis of own-body perception. Front. Hum. Neurosci. 24:141 10.3389/fnhum.2015.00141 PMC4371812 25852524

[B37] Land W. M. Volchenkov D. Bläsing B. Schack T. (2013). From action representation to action execution: exploring the links between cognitive and biomechanical levels of motor control. Front. Comput. Neurosci. 7:127. 10.3389/fncom.2013.00127 24065915 PMC3776155

[B38] Law B. Post P. McCullagh P. (2017). Modeling in Sport and Performance. Oxford Research Encyclopedia of Psychology. 10.1093/acrefore/9780190236557.013.159

[B39] Lenggenhager B. Mouthon M. Blanke O. (2009). Spatial aspects of bodily self-consciousness. Consciousness Cogn. 18, 110–117. 10.1016/j.concog.2008.11.003 19109039

[B40] Longo M. R. Haggard P. (2009). Sense of agency primes manual motor responses. Perception 38, 69–78. 10.1068/p6045 19323137

[B41] Magill R. A. (2001). Augmented feedback in motor skill acquisition, in Handbook of Sport Psychology, eds Singer R. N. Hausenblas H. A. Janelle C. M (New York, NY: Wiley, 86–114.

[B42] Magill R. A. Anderson D. I. (2012). The roles and uses of augmented feedback in motor skill acquisition, in Skill Acquisition in Sport: Research, Theory and Practice, eds Hodges N. J. Williams A. M (New York, NY: Routledge, 3–21.

[B43] Marschall F. Bund A. Wiemeyer J. (2007). Does frequent augmented feedback really degrade learning? A meta-analysis. Bewegung Training 1, 74–85.

[B44] Martens R. Burwitz L. Zuckerman J. (1976). Modeling effects on motor performance. Research Quarterly. Am. Alliance Health Phys. Educ. Recreat. 47, 277–291. 10.1080/10671315.1976.10615372 1067645

[B45] Maselli A. Slater M. (2013). The building blocks of the full body ownership illusion. Front. Hum. Neurosci. 7:83. 10.3389/fnhum.2013.00083 23519597 PMC3604638

[B46] McCullagh P. Law B. Ste-Marie D. (2012). Modeling and performance, in The Oxford Handbook of Sport and Performance Psychology, ed Murphy S. M (Oxford: University Press), 250–272. 10.1093/oxfordhb/9780199731763.013.0013

[B47] Mervis C. B. Rosch E. (1981). Categorization of natural objects. Ann. Rev. Psychol. 32, 89–113. 10.1146/annurev.ps.32.020181.000513

[B48] Miles H. C. Pop S. R. Watt S. J. Lawrence G. P. John N. W. (2012). A review of virtual environments for training in ball sports. Computers & Graphics 36, 714–726. 10.1016/j.cag.2012.04.007

[B49] Neumann D. L. Moffitt R. L. Thomas P. R. Loveday K. Watling D. P. Lombard C. L. (2018). A systematic review of the application of interactive virtual reality to sport. Virtu. Real. 22, 183–198. 10.1007/s10055-017-0320-5

[B50] Ossmy O. Mukamel R. (2016). Neural network underlying intermanual skill transfer in humans. Cell Rep. 17, 2891–2900. 10.1016/j.celrep.2016.11.009 27974204

[B51] Ossmy O. Mukamel R. (2017a). Short term motor-skill acquisition improves with size of self-controlled virtual hands. PLoS ONE 12:e0168520. 10.1371/journal.pone.0168520 28056023 PMC5215900

[B52] Ossmy O. Mukamel R. (2017b). Using virtual reality to transfer motor skill knowledge from one hand to another. J. Visual. Exp. 127:55965 10.3791/55965 PMC5752261 28994768

[B53] Ossmy O. Mukamel R. (2018). Perception as a route for motor skill learning: perspectives from neuroscience. Neuroscience 382, 144–153. 10.1016/j.neuroscience.2018.04.016 29694916

[B54] Rizzolatti G. Fogassi L. Gallese V. (2001). Neurophysiological mechanisms underlying the understanding and imitation of action. Nat. Rev. Neurosci. 2, 661–670. 10.1038/35090060 11533734

[B55] Rizzolatti G. Sinigaglia C. (2016). The mirror mechanism: a basic principle of brain function. Nat. Rev. Neurosci. 17, 757–765. 10.1038/nrn.2016.135 27761004

[B56] Rohbanfard H. Proteau L. (2011). Learning through observation: a combination of expert and novice models favors learning. Exp. Brain Res. 215, 183–197. 10.1007/s00221-011-2882-x 21986667

[B57] Rosch E. (1978). Principles of categorization, in Cognition and Categorization, eds Rosch E. Loyd B. B (Hillsdale: Erlbaum, 27–48.

[B58] Rosch E. Mervis D. B. (1975). Family resemblances: studies in the internal structure of categories. Cogn. Psychol. 7, 573–605. 10.1016/0010-0285(75)90024-9

[B59] Salamin P. Tadi T. Blanke O. Vexo F. Thalmann D. (2010). Quantifying effects of exposure to the third and first-person perspectives in virtual-reality-based training. IEEE Trans. Learn. Technol. 3, 272–276. 10.1109/TLT.2010.13

[B60] Salmoni A. W. Schmidt R. A. Walter C. B. (1984). Knowledge of results and motor learning: a review and critical reappraisal. Psychol. Bull. 95, 355–386. 10.1037/0033-2909.95.3.355 6399752

[B61] Santos J. M. Embrechts M. (2009). On the use of the adjusted rand index as a metric for evaluating supervised classification, in Artificial Neural Networks - ICANN, Lecture Notes in Computer Science, eds Alippi C. Polycarpou M. Panayiotou C. Ellinas G (Berlin: Springer), 175–184. 10.1007/978-3-642-04277-5_18

[B62] Schack T. (2004). The cognitive architecture of complex movement. Int. J. Sport Exerc. Psychol. 2, 403–438. 10.1080/1612197X.2004.9671753

[B63] Schack T. (2012). Measuring mental representations, in Measurement in Sport and Exercise Psychology, eds Tenenbaum G. Eklund R. C. Kamata A (Champaign: Human Kinetics, 203–214.

[B64] Schack T. Mechsner F. (2006). Representation of motor skills in human long-term memory. Neurosci. Lett. 391, 77–81. 10.1016/j.neulet.2005.10.009 16266782

[B65] Schack T. Ritter H. (2013). Representation and learning in motor action: bridges between experimental research and cognitive robotics. New Ideas Psychol. 31, 258–269. 10.1016/j.newideapsych.2013.04.003

[B66] Schmidt R. Lee T. (2011). Motor Control and Learning: A Behavioral Emphasis. Champaign: Human Kinetics.

[B67] Schmidt R. A. (1991). Frequent augmented feedback can degrade learning: evidence and interpretations, in NATO ASI Series; Series D: Behavioral and Social Sciences, Vol. 62. *Tutorials in Motor Neuroscience*, eds Requin J. Stelmach G. E (New York, NY: Kluwer Academic/Plenum Publishers), 59–75. 10.1007/978-94-011-3626-6_6

[B68] Schmidt R. A. Young D. E. Swinnen S. Shapiro D. C. (1989). Summary knowledge of results for skill acquisition: Support for the guidance hypothesis. J. Exp. Psychol. Learn. Memory Cogn. 15, 352–359. 10.1037//0278-7393.15.2.352 2522520

[B69] Scully D. M. Newell K. M. (1985). Observational learning and the acquisition of motor skills: Towards a visual perception perspective. J. Hum. Mov. Stud. 11, 169–186.

[B70] Senna I. Russo C. Parise C. V. Ferrario I. Bolognini N. (2015). Altered visual feedback modulates cortical excitability in a mirror-box-like paradigm. Exp. Brain Res. 233,1921–1929. 10.1007/s00221-015-4265-1 25850405

[B71] Shea C. H. Wulf G. (1999). Enhancing motor learning through external-focus instructions and feedback. Hum. Mov. Sci. 18, 553–571. 10.1016/S0167-9457(99)00031-7

[B72] Sigrist R. Rauter G. Marchal-Crespo L. Riener R. Wolf P. (2015). Sonification and haptic feedback in addition to visual feedback enhances complex motor task learning. Exp. Brain Res. 233, 909–925. 10.1007/s00221-014-4167-7 25511166

[B73] Sigrist R. Rauter G. Riener R. Wolf P. (2013). Augmented visual, auditory, haptic, and multimodal feedback in motor learning: a review. Psychonom. Bull. Rev. 20, 21–53. 10.3758/s13423-012-0333-8 23132605

[B74] Slater M. Spanlang B. Sanchez-Vives M. Blanke O. (2010). First person experience of body transfer in virtual reality. PLoS ONE 5:e10564. 10.1371/journal.pone.0010564 20485681 PMC2868878

[B75] Sveistrup H. (2004). Motor rehabilitation using virtual reality. J. NeuroEngineering and Rehabil. 1, 10. 10.1186/1743-0003-1-10 15679945 PMC546406

[B76] Tang R. Yang X. D. Bateman S. Jorge J. Tang A. (2015). Physio@home: exploring visual guidance and feedback techniques for physiotherapy exercises,in Proceedings of the 33rd Annual ACM Conference on Human Factors in Computing Systems, ACM, 4123–4132. 10.1145/2702123.2702401

[B77] Todorov E. Shadmehr R. Bizzi E. (1997). Augmented feedback presented in a virtual environment accelerates learning of a difficult motor task. J. Motor Behav. 29, 147–158. 10.1080/00222899709600829 12453791

[B78] Tsakiris M. Longo M. R. Haggard P. (2010). Having a body versus moving your body: neural signatures of agency and body-ownership. Neuropsychologia 48, 2740–2749. 10.1016/j.neuropsychologia.2010.05.021 20510255

[B79] Tsakiris M. Prabhu G. Haggard P. (2006). Having a body versus moving your body: how agency structures body ownership. Conscious. Cogn. 15 423–432. 10.1016/j.concog.2005.09.004 16343947

[B80] Waltemate T. Hülsmann F. Pfeiffer T. Kopp S. Botsch M. (2015). Realizing a low-latency virtual reality environment for motor learning,in Proceedings of the 21st ACM Symposium on Virtual Reality Software and Technology, ACM, 139–147. 10.1145/2821592.2821607

[B81] Waltemate T. Senna I. Hülsmann F. Rohde M. Kopp S. Ernst M. (2016). The impact of latency on perceptual judgments and motor performance in closed-loop interaction in virtual reality, in Proceedings of the 22nd ACM Conference on Virtual Reality Software and Technology, ACM, 27–35. 10.1145/2993369.2993381

[B82] Winstein C. J. Schmidt R. A. (1990). Reduced frequency of knowledge of results enhances motor skill learning. J. Exp. Psychol. Learn. Memory Cogn. 16, 677–691. 10.1037//0278-7393.16.4.677

