# Supplementary material for: Superimposed Skilled Performance in a Virtual Mirror Improves Motor Performance and Cognitive Representation of a Full Body Motor Action
Source: Front Robot AI. 2019 Jun 21;6:43. doi: 10.3389/frobt.2019.00043 (PMC7805859; doi:10.3389/frobt.2019.00043)
Supplement: Supplementary file 2 [file Data_Sheet_1.pdf]

Supplementary material

Superimposed Skilled Performance in a Virtual Mirror Improves Motor Performance and Cognitive Representation of a Full Body Motor Action

Felix Hülsmann, Cornelia Frank, Irene Senna, Marc O. Ernst, Thomas Schack and Mario Botsch

Motor Performance Measures

This supplement describes the parameters that we used to measure motor performance.

Dynamic Time Warping (DTW)

DTW aligns two time series, in our case the participant's performance and the skilled performance via calculating the optimal match between them. That means for each frame in the participant's movement, DTW calculates at least one corresponding frame in the skilled movement and vice-versa (Müller, 2007).

We performed DTW based on the joint positions. We used all joints, but the root joint, as well as three joints in the back (spine markers placed at l2, t5, and t10). We excluded these joints as we wanted to mainly focus on the movement of the extremities and the joints in the back tended to induce a high level of noise in our setup. DTW in general works as follows: Let $\mathrm{T}_{participant}$, $\mathrm{T}_{\mathrm{skilled}}$ be two motion capture trajectories which consist of n and m successive postures. $T_{participant}$ is the movement performed by the participant, $T_{skilled}$ is the skilled movement. Each posture of a trajectory consists of k translations, one for each tracked joint. For instance, $\boldsymbol{x}_d$ denotes the translation of joint d. To perform DTW, a $\mathrm{n} \times \mathrm{m}$ local cost matrix $\mathbf{M}$ is constructed. Each element (i, j) of this matrix corresponds to the distances between the postures $\mathrm{T}_{participant}(\mathrm{i})$ and $\mathrm{T}_{skilled}(\mathrm{j})$:

$$\mathbf{M}(\mathrm{i}, \mathrm{j}) = \sum_{\mathrm{d}=1}^{\mathrm{k}} ||\boldsymbol{x}_d(i) - \boldsymbol{x}_d(j)||.$$

Based on dynamic programming, we determined an optimal path of corresponding frames through this matrix according to (Müller, 2007).

We extracted two features based on DTW: the temporal as well as the spatial error. The temporal error was calculated as follows: For each frame in a participant's movement, we

calculated the change in the temporal offset from the performed movement to the skilled movement. Example: If frame 200 of the participant's movement mapped to frame 210 of the skilled movement and frame 201 of the participant's movement mapped to frame 215, the error at frame 201 was -4. Finally, we returned the RMSE of these shifts. To be more specific, we performed the following calculation:

$$error_{temporal} = \left(\frac{1}{|T_{participant}|} \sum_{f=1}^{|T_{participant}|} \left((f - w(f)) - ((f-1) - w(f-1)) \right)^2 \right)^{1/2}.$$

Here, $w(f)$ is the frame number of the skilled movement that was mapped on frame number f of the participant's movement according to the frame-wise correspondences calculated by DTW. If, according to the optimal path, multiple frames of the skilled movement mapped on the same frame of the participant's movement, we selected the one that is in the middle of these frames on the temporal axis. The spatial error was the averaged value of $\boldsymbol{M}$ on the optimal path:

$$error_{spatial} = \frac{1}{|path|} \sum_{p=1}^{|path|} \boldsymbol{M}(path(p)).$$

Here, $path$ specifies the optimal path through $\boldsymbol{M}$ that was calculated by DTW. Each entry p is a tuple $(i, j) \epsilon \; path$ that contains the frame numbers i and j of the trajectories $\mathrm{T}_{participant}$ and $\mathrm{T}_{\mathrm{skilled}}$ that correspond to each other, i.e., that lie on the optimal path. $|path|$ denotes the length of the optimal path. See (Müller, 2007) for a formal definition of $path$.

Center of mass at the deepest point

We estimated a simplified center of mass based on the centroid of the joint positions. More specifically, the center of mass of the participant's trajectory at the deepest point of the squat was calculated as follows:

$$\boldsymbol{com} = \frac{1}{k}\sum_{d=1}^{k} \mathbf{x}_{\mathrm{d}}(f_{deepest}).$$

Here, k denotes the number of joints and $\boldsymbol{x}_d(f_{deepest})$ denotes the translation of joint d at the deepest point (frame $f_{deepest}$) of the squat.

Principal Component Analysis

Principal Component Analysis (PCA) is typically used in the field of dimensionality reduction (Bishop, 2006). For a given data set, it searches for a set of linear combinations that capture a given amount of variance inside the data. It reduces the high-dimensional data set into a smaller number of structural components. We determined the number of principal components needed to cover 85% of the variance inside our data for each participant and each test phase (pre-test, post-test, retention-test). To focus only on the spatial properties, we first performed DTW between each trajectory of a participant T_i and the first trajectory of this participant in the given phase T_0. We used the correspondence path determined by DTW to warp each movement into the timing of T_0: For each frame of T_i, the corresponding frame in T_0 was extracted. Next, we constructed a feature vector that consisted of the joint translations of these frames. This vector had the length $3k|T_0|$, where k was the number of joints. $|T_0|$ was the length of trajectory T_0. Then we calculated the PCA based on the feature vectors for each participant and the test phases (pre-test, post-test, retention-test).

Supplementary References

Bishop, C. M. (2006). *Pattern Recognition and Machine Learning*. New York, NY: Springer Science and Business Media.
